# Comparative efficacy of interventional therapy with or without targeted immunotherapy in Child-Pugh B hepatocellular carcinoma patients: a single-center, retrospective study

**DOI:** 10.3389/fonc.2025.1541805

**Published:** 2025-05-01

**Authors:** Xu-Wei Guo, Man Zhao, Xiao-Ling Duan, Guang-Jie Han, Jin-Feng Wang, Jian-Fei Shi, Xin Han, Fei Yin, Guang Yang

**Affiliations:** ^1^ Department of Gastroenterology, The Fourth Hospital of Hebei Medical University, Shijiazhuang, Hebei, China; ^2^ Department of Interventional Radiology, The Fourth Hospital of Hebei Medical University, Shijiazhuang, Hebei, China

**Keywords:** hepatocellular carcinoma, Child-Pugh B, interventional therapy, immune checkpoint inhibitors, tyrosine kinase inhibitors

## Abstract

**Background:**

Current large clinical trials mainly focus on Child-Pugh A (CP-A) stage hepatocellular carcinoma (HCC) patients, with limited data on CP-B patients especially those classified as B8-9, whose treatment needs remain inadequately addressed. This study aims to evaluate the safety efficacy of interventional treatments, with or without targeted-immunotherapy and characteristics of CP-B stage HCC patients receiving.

**Methods:**

This single-center retrospective investigation incorporated 119 patients were stratified into two cohorts: the interventional therapy cohort (42) and the combined targeted immunotherapy cohort (77). The clinical data, overall survival (OS), progression-free survival (PFS), and therapeutic efficacy of both groups were meticulously recorded and comprehensively analyzed. Survival disparities were statistically compared employing the Kaplan-Meier survival analysis method and the log-rank test. Tumor remission was appraised in accordance with the RECIST 1.1 and mRECIST criteria. Independent influencing factors were discerned through multifactorial COX regression analysis. Subsequently, survival prediction models were constructed to generate column line graphs, and the safety profiles and adverse events associated with diverse treatment modalities were also evaluated.

**Results:**

119 patients with CP-B grade HCC were included, and the median survival (mOS) of patients who received combination therapy was 21.4 months (vs 13.2, P=0.038) superior to that of interventional therapy, and the median progression-free survival (mPFS) of 12.7 months (vs 10.9 months, P=0.183) was not significantly improved. The OS of patients in group B7 who received combination therapy was 24.6 months (vs 11.9, P=0.006) was superior to that of the intervention, while there was no significant improvement in patients in groups B8-9. The objective remission rate (ORR) was higher in the combination therapy than in the intervention group (RECIST: 32.5% vs 11.9%, P = 0.014; mRECIST: 48.1% vs 23.8%, P = 0.010). Except for Child-Pugh score progression (P = 0.003), there was no significant difference in the occurrence of all-grade and ≥grade 3 adverse events in the combination therapy group compared with the intervention group (P > 0.05).

**Conclusion:**

Interventional therapy combined with targeted and immunotherapy can be a safe and effective treatment for patients with Child-Pugh grade B hepatocellular carcinoma in the setting of controlled liver function impairment.

## 1 Introduction

According to global cancer registry data, liver cancer is the sixth most common malignant tumor worldwide and has the third highest mortality rate. In Asia, particularly in China, the incidence of liver cancer is the highest, accounting for 42.5% of cases globally (1). Hepatocellular carcinoma (HCC) and intrahepatic cholangiocarcinoma (ICC) are the two main histological subtypes of primary liver cancer. HCC accounts for 80% of all primary liver cancer cases globally (2), and most HCC cases occur in patients with liver cirrhosis, with no significant gender differences observed (3). The incidence of HCC is notably high in both Asia and Africa. Liver function reserve is a crucial factor influencing the prognosis and treatment decisions for HCC patients, particularly in determining targeted therapies for those with advanced HCC (4). The Child-Pugh grades (CP) is an important tool for assessing liver function in patients with cirrhosis, including A, B, and C. The updated guidelines from the American Society of Clinical Oncology (ASCO) in 2024 indicate (5) that systemic therapies such as atezolizumab, bevacizumab, and tyrosine kinase inhibitors can be as first-line treatment options for advanced HCC patients with Child-Pugh A (CP-A). However, for Child-Pugh B (CP-B) patients, a cautious treatment options should be considered due to impaired liver function (6). Child-Pugh C (CP-C) advanced HCC patients typically have severely compromised liver function and are usually limited to conservative or palliative care. Given the complexity and heterogeneity of CP-B HCC patients, research into individualized and precise treatment strategies for this population is particularly important. Clinically, it is essential to consider various factors, including the liver function (7), tumor characteristics, and overall health status, while also monitoring changes in liver function and treatment-related adverse events to make timely adjustments to the treatment plan.

The systemic treatment for intermediate and advanced HCC has entered an era of combination therapy, driven by ongoing clinical research. The use of immune checkpoint inhibitors (ICIs), targeted therapies, and local treatments, either individually or in combination, is increasingly reported in CP-B HCC patients. The exploration of diverse treatment combinations with ICIs - based systemic therapy serving as the cornerstone regimen has ushered in novel strategies for the treatment management of HCC patients (8). The mechanisms underlying the synergistic anti - tumor effects achieved by combining systemic therapy with local Trans - arterial chemoembolization (TACE) treatment have been extensively explored and validated. The principle of combining immunotherapy, targeted therapy, and interventional treatments is becoming a trend in the management of HCC (9). Interventional treatments can enhance immunogenicity by inducing the release of tumor antigens and modifying the tumor microenvironment, making tumor cells more sensitive to both immune and targeted therapies, thus improving treatment outcomes (10, 11). Recent clinical studies (12–14) have demonstrated that the combination of these three approaches shows significantly better efficacy than interventional treatments alone. Some studies (15) have focused on specific populations, such as patients with HCC and portal vein tumor thrombosis (PVTT), showing that those receiving combination therapy experience higher tumor response rates, longer survival, and better progression-free survival compared to those undergoing trans–arterial chemoembolization (TACE) alone. For CP-B HCC patients, this combination therapy approach may address the shortcomings of sole interventional treatment by improving therapeutic efficacy while minimizing damage to liver function, thus providing new avenues for improving patient prognosis. However, research comparing the outcomes of immune and targeted combination therapy with standard interventional treatment in CP-B HCC patients is still limited. This retrospective study aims to analyze these comparisons and develop predictive models to evaluate prognostic factors, enabling further stratification of CP-B HCC patients.

## 2 Materials and methods

### 2.1 Study population

The retrospective follow-up study includes clinical data from patients with CP-B HCC patients and underwent interventional treatment at the Fourth Hospital of Hebei Medical University from 2019-01-01 to 2022-05-01. Inclusion criteria (1): Age≥18 years; (2) Diagnosed with intermediate to advanced HCC through imaging and/or histopathology; (3) Classified as Child-Pugh B; (4) Received interventional treatment (e.g., TACE) either alone or in combination with targeted or immunotherapy. Exclusion criteria: (1) Child-Pugh classification A or C; (2) Presence of other malignant tumors; (3) Incomplete case data or follow-up information. This study has been approved by the Ethics Committee of the Fourth Hospital of Hebei Medical University.

### 2.2 Child-Pugh B grade

Based on the Child-Pugh scoring system, we assessed patients for ascites, hepatic encephalopathy, albumin levels, total bilirubin, and prothrombin time using their laboratory results. We recorded the scores of patients first classified as B and analyzed their baseline scores along with subsequent treatment choices and survival outcomes.

### 2.3 Interventional treatment, targeted treatment and immunotherapy treatment

The interventional treatment methods included TACE, hepatic arterial infusion chemotherapy (HAIC), or the combination of both. Patients may also receive systemic therapies such as targeted therapy (including sorafenib, lenvatinib, apatinib, regorafenib) and immunotherapy (including sintilimab, camrelizumab, pembrolizumab, toripalimab). We tracked the treatment regimens patients received after being classified as Child-Pugh B through medical records and follow-up, categorizing them into interventional treatment and combination therapy groups based on whether they received additional systemic therapy.

### 2.4 Study outcomes

(1) Survival status: We recorded the current survival status of patients based on inpatient, outpatient follow-up results and telephone follow-ups. (2) Baseline and efficacy evaluation: Baseline data were collected from the laboratory results and clinical results of enrolled CP-B HCC patients at the initial assessment. Tumor treatment efficacy was evaluated using the Response Evaluation Criteria in Solid Tumors (RECIST 1.1) and its modified version (mRECIST). Tumor responses were assessed 3-4 weeks post-treatment using enhanced CT or MRI and categorized into four levels: complete response (CR), partial response (PR), stable disease (SD), and progressive disease (PD). (3) We evaluated treatment-related adverse events using the Common Terminology Criteria for Adverse Events (CTCAE) version 5.0.

### 2.5 Following

After inclusion in the study, patients were followed up through inpatient and outpatient visits and telephone contacts, with the follow-up period ending on 2024-05-01, or at the time of patient death.

### 2.6 Statistical analysis

Statistical analyses were performed using IBM SPSS Statistics 27 and GraphPad Prism 10.1.2 software. A P-value of <0.05 was considered statistically significant. Continuous variables were analyzed using independent samples t-tests or Mann-Whitney U tests, while categorical variables were analyzed using chi-square tests, Fisher’s exact test, or Mann-Whitney U tests as appropriate. Kaplan-Meier survival analysis was conducted, with differences in survival compared using log-rank tests. Variables with P < 0.05 from univariate and multivariate Cox regression analyses were included in the clinical prediction model for survival in CP-B HCC patients. A visual nomogram was constructed, and the area under the curve (AUC) was calculated to assess the model’s discriminative ability. Calibration curves were used to evaluate the calibration accuracy of the model, and decision curve analysis (DCA) was employed to assess its clinical utility (16).

## 3 Results

### 3.1 Baseline characteristics

A total of 119 patients were included in the study, with 42 patients (35.3%) in the interventional treatment group (IT) and 77 patients (64.7%) in the combination treatment group (CT). Among these, 72 patients (60.5%) had a Child-Pugh score of B7, 31 patients (26.1%) had a score of B8, and 16 patients (13.4%) had a score of B9. A history of hepatitis B virus (HBV) or hepatitis C virus (HCV) infection was present in 95 patients (79.8%), while 24 patients (20.2%) had no viral infections. PVTT was observed in 39 patients (32.0%). According to the Barcelona Clinic Liver Cancer (BCLC) staging, there were 21 patients (17.6%) in stage A, 43 patients (36.1%) in stage B, and 55 patients (46.2%) in stage C. Based on the China Liver Cancer Staging (CNLC), 42 patients (36.1%) were in stages I–IIb, 23 patients (19.3%) in stage IIIa, and 53 patients (44.5%) in stage IIIb. Among the patients, 104 had liver cirrhosis, while 5 did not. Comparisons between the interventional treatment group and the combination therapy group showed no statistically significant differences in terms of gender, age, Eastern Cooperative Oncology Group performance status (ECOG), initial treatment status, ascites, Child-Pugh score, albumin-bilirubin grade (ALBI), BCLC stage, CNLC stage, PVTT, etiology, extrahepatic metastasis, number of tumors, tumor diameter (10 cm as the cutoff), number of interventional procedures, and presence of cirrhosis (P > 0.005) in **Table 1**.

**Table 1:** Baseline characteristics.

Variables	Total (n=119)	IT (n=42)	CT (n=77)	P
**Sex**				0.269
male	97 (81.5)	32 (76.2)	65 (84.4)	
female	22 (18.5)	10 (23.8)	12 (15.6)	
**Age**	59.2±10.7	58.3±10.2	60.7±11.5	0.253
**ECOG**				0.487
0	73 (61.3)	24 (57.1)	49 (63.6)	
1	46 (38.7)	18 (42.9)	28 (36.4)	
**Treatment history**				0.254
no	74 (62.2)	29 (69.0)	45 (58.4)	
yes	45 (37.8)	13 (31.0)	32 (41.6)	
**Ascites**				0.964
no	57 (47.9)	20 (47.6)	37 (48.1)	
yes	62 (52.1)	22 (52.4)	40 (51.9)	
**Child-Pugh score**				0.659
7	72 (60.5)	24(57.1)	48(62.3)	
8	31 (26.1)	13(31.0)	18(23.4)	
9	16 (13.4)	5(11.9)	11(14.3)	
**Child-Pugh**				0.580
B7	72 (60.5)	24 (57.1)	48 (62.3)	
B8-9	47 (39.5)	18 (42.9)	29 (37.7)	
**ALBI**				0.757
1	6 (5.0)	3 (7.1)	3 (3.9)	
2	104 (87.4)	36 (85.7)	68 (88.3)	
3	9 (7.6)	3 (7.1)	6 (7.8)	
**BCLC**				0.417
A	21 (17.6)	5 (11.9)	16 (20.8)	
B	43 (36.1)	15 (35.7)	28 (36.4)	
C	55 (46.2)	22 (52.4)	33 (42.9)	
**CNLC**				0.382
Ia/Ib/IIa/IIb	43 (36.1)	13(31.0)	30(39.0)	
IIIa	23 (19.3)	7(16.7)	16(20.8)	
IIIb	53 (44.5)	22(52.4)	31(40.3)	
**up to 7**				0.083
no	39 (32.8)	18 (42.9)	21 (27.3)	
yes	80 (67.2)	24 (57.1)	56 (72.7)	
**PVTT**				0.849
no	78 (65.5)	28 (66.7)	50 (64.9)	
yes	41 (34.5)	14 (33.3)	27 (35.1)	
**HBV/HCV**				0.092
no	24 (20.2)	12 (28.6)	12 (15.6)	
yes	95 (79.8)	30 (71.4)	65 (84.4)	
**Extrahepatic metastases**				0.438
no	68 (57.1)	22 (52.4)	46 (59.7)	
yes	51 (42.9)	20 (47.6)	31 (40.3)	
**Tumor number**				0.821
≤2	55 (46.2)	20 (47.6)	35 (45.5)	
>2	64 (53.8)	22 (52.4)	42 (54.5)	
**Tumor diameter**				0.569
≤10cm	84 (70.6)	31 (73.8)	53(68.8)	
>10cm	35 (29.4)	11(26.2)	24(31.2)	
**Tumor diameter**				0.054
≤5cm	43 (36.1)	20 (47.6)	23 (29.9)	
>5cm	76 (63.9)	22 (52.4)	54 (70.1)	
**Times of intervention**				0.124
≤3	80 (67.2)	32 (76.2)	48 (62.3)	
>3	39 (32.8)	10 (23.8)	29 (37.7)	
**Cirrhosis**				0.683
no	15 (12.6)	6 (14.3)	9 (11.7)	
yes	104 (87.4)	36 (85.7)	68 (88.3)	
**Smoking**				0.802
no	52 (43.7)	19 (45.2)	33 (42.9)	
yes	67 (56.3)	23 (54.8)	44 (57.1)	
**Alcohol**				0.121
no	68 (57.1)	20 (47.6)	48 (62.3)	
yes	51 (42.9)	22 (52.4)	29 (37.7)	
**Hypertension**	44 (37.0)	18 (42.9)	26 (33.8)	0.326
**Cardiovascular disease**	6 (5.0)	2 (4.8)	4 (5.2)	1.000
**Diabetes**	21 (17.6)	5 (11.9)	16 (20.8)	0.225
**AFP** **(<1210ng/ml VS ≥1210ng/ml)**				0.118
<1210 (ng/ml)	86 (72.3)	34 (81.0)	52 (67.5)	
≥1210 (ng/ml)	33 (27.7)	8 (19.0)	25 (32.5)	
**AFP** **(<400ng/ml VS ≥400ng/ml)**				0.009
<400 (ng/ml)	75 (63.0)	33 (78.6)	42 (54.5)	
≥400 (ng/ml)	44 (37.0)	9 (21.4)	35 (45.5)	
**ALT (U/L)**	32.2 (21.3-52.6)	28.2 (20.2-38.2)	37.0 (22.5-56.3)	0.595
**AST (U/L)**	55.5 (35.5-85.6)	46.7 (34.6-62.7)	61.3 (36.0-89.2)	0.278
**TBIL (μmol/L)**	24.3(15.8-37.7)	24.5(15.6-36.7)	24.3(16.2-37.9)	0.438
**Albumin**				0.944
normal	43 (36.1)	15 (35.7)	28 (36.4)	
low	76 (63.9)	27 (64.3)	49 (63.6)	
**NLR**	3.0 (2.0-5.0)	2.6 (1.8-4.3)	3.1 (2.1-5.3)	0.363
**PLR**	115.3 (72.6-165.7)	118.9 (63.8-180.1)	112.8 (80.7-163.9)	0.874
**PT**	14.1 (12.4-15.3)	14.2 (12.2-15.8)	14.1 (12.6-15.0)	0.633
**D-dimer**	0.447 (0.225-0.901)	0.377 (0.212-0.758)	0.550 (0.238-0.927)	0.316

IT, interventional treatment; CT, combination treatment. Continuous variables are expressed as mean ± standard deviation (Mean ± SD) or median and interquartile range, denoted as M(Q1, Q3). Categorical variables are represented as frequency and percentage, denoted as n (%). ECOG, Eastern Cooperative Oncology Group; BCLC, Barcelona Clinic Liver Cancer; CLNC, China Liver Cancer; up to 7 criterion, the sum of the number of liver cancer tumors and their diameters exceeds 7; PVTT, portal vein tumor thrombosis; ALT, alanine aminotransferase; AST, aspartate aminotransferase; TBIL, total bilirubin, ALBI score is the albumin-bilirubin score, calculated based on albumin (ALB) and total bilirubin (TB), ALBI=0.66×log10[TB (μmol/L)]-0.085×[ALB (g/L)], Grade 1≤-2.60, -2.60<Grade 2≤-1.39, Grade 3>-1.39; CNLC staging is the Chinese liver cancer staging; AFP is alpha-fetoprotein; NLR is neutrophil count/lymphocyte count; PLR is platelet count/lymphocyte count; PT, prothrombin time.

### 3.2 Survival results

#### 3.2.1 All patients

**Figure 1:**
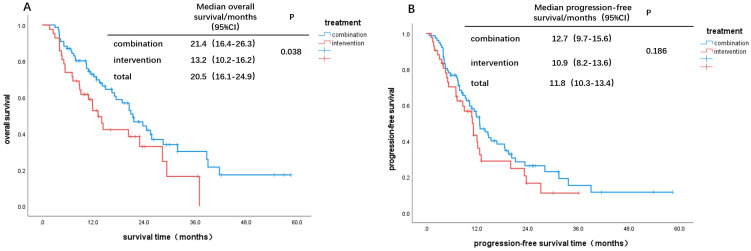
Kaplan–Meier curves for OS and PFS: **(A)** OS of all patients, **(B)** PFS of all patient.

As of the end of the follow-up, the survival outcomes for the 119 patients were as follows: the median overall survival (mOS) for the CT group was 21.4 months, while the mOS for the IT group was 13.2 months. Kaplan-Meier survival analysis and log-rank tests indicated a statistically significant difference in survival between the two groups (χ² = 4.295, P = 0.038, **Figure 1A**), with the CT group showing superior survival benefits compared to the IT group. The median progression-free survival (mPFS) for the IT group was 12.7 months, compared to 10.9 months for the CT group; however, this difference was not statistically significant (χ² = 1.753, P = 0.186, **Figure 1B**).

#### 3.2.2 Different Child-Pugh score

**Figure 2:**
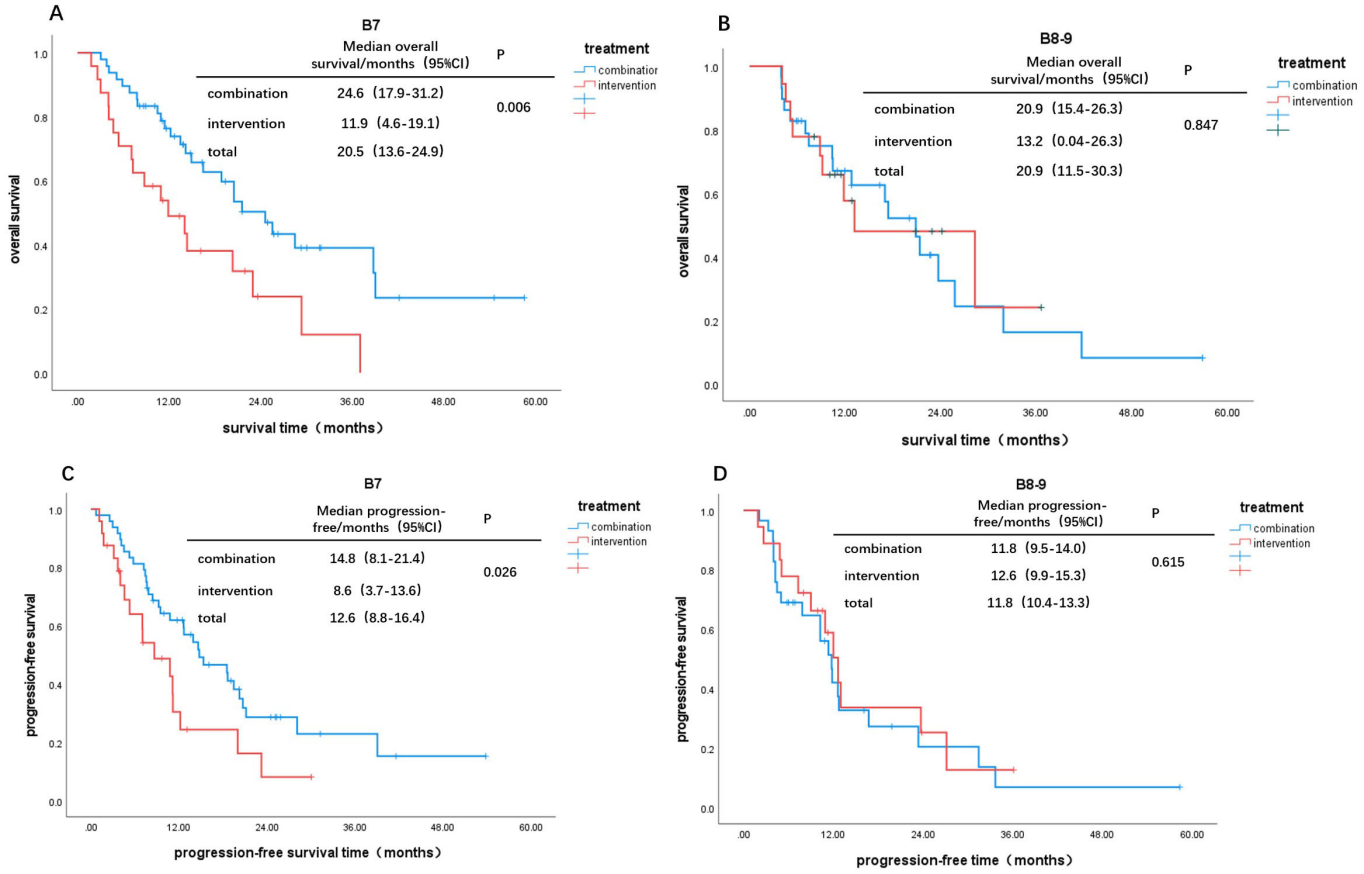
Kaplan-Meier curves for different Child-Pugh scores: OS: **(A)** B7, **(B)** B8-9; PFS: **(C)** B7, **(D)** B8-9.

**Figure 3:**
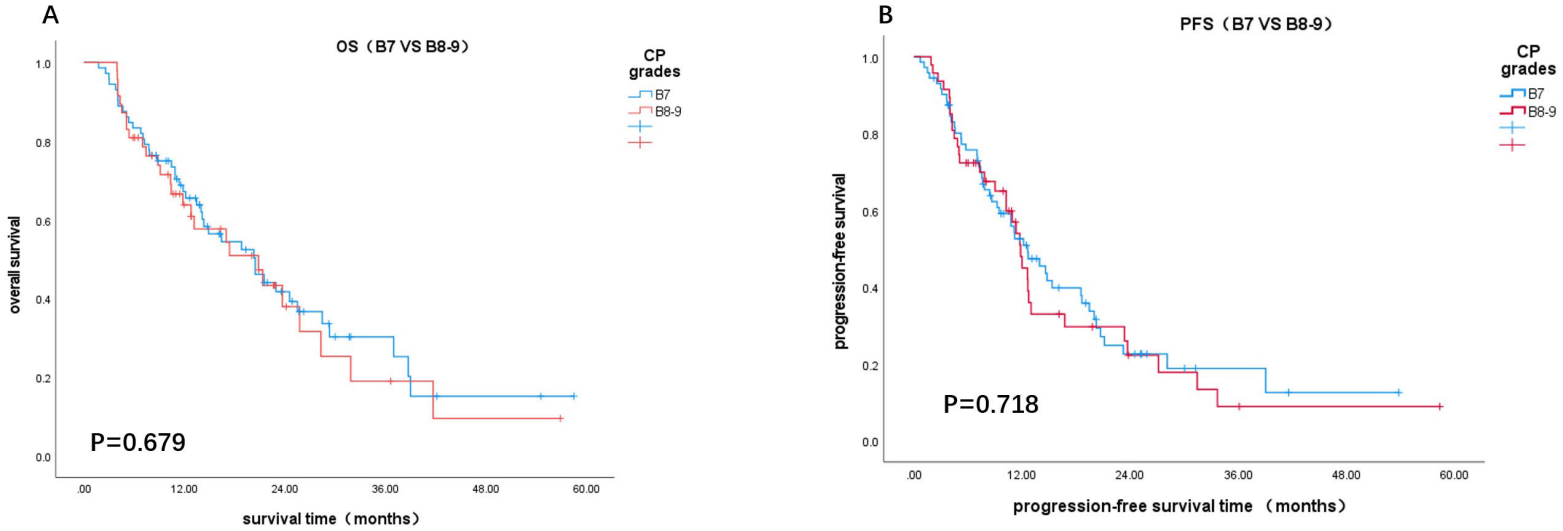
Kaplan-Meier curves for B7 vs. B8-9: **(A)** OS (A), **(B)** PFS.

Based on Child-Pugh scores of 7 and 8-9, patients were divided into B7 and B8-9 groups. When comparing the IT group to the CT group, the OS and PFS in the B7 group showed statistically significant differences (χ² = 7.578, P = 0.006; χ² = 4.953, P = 0.026, **Figures 2A, C**). In contrast, there were no statistically significant differences in OS and PFS between the two groups in the B8-9 group (χ² = 0.037, P = 0.847; χ² = 0.252, P = 0.615, **Figures 2B, D**). For patients with a Child-Pugh score of 7, the CT group demonstrated better survival outcomes than the IT group. However, for scores of 8 and 9, there was no significant difference in survival benefits between the two groups. Furthermore, comparisons of survival outcomes between the B7 and B8-9 groups indicated no significant statistical differences in OS and PFS (χ² = 0.172, P = 0.679; χ² = 0.131, P = 0.718, **Figures 3A, B**).

### 3.3 Tumor response

According to the RECIST 1.1 criteria, one patient (0.8%) was assessed as having CR, 29 patients (24.4%) had PR, 85 patients (71.4%) had SD, and 4 patients (3.4%) had PD. The rates of CR (1.3% vs. 0%), PR (31.2% vs. 11.9%), objective response rate (ORR) (32.5% vs. 11.9%), and disease control rate (DCR) (98.7% vs. 92.9%) were all higher in the CT group compared to the IT group. The difference in ORR between the two groups was statistically significant (P = 0.014), while the DCR showed no significant difference (P = 0.247). According to the mRECIST criteria, the CT group also had higher rates of CR (7.8% vs. 0%), PR (40.3% vs. 23.8%), and ORR (48.1% vs. 23.8%) compared to the IT group, with the difference in ORR being statistically significant (P=0.010) (**Table 2**).

**Table 2:** Tumor treatment effect.

Effect	RECIST 1.1				mRECIST			
	Total (n=119)	Intervention (n=42)	Combination (n=77)	P value	total (n=119)	Intervention (n=42)	Combination (n=77)	P value
Best effect				**0.03**				**0.002**
CR	1 (0.8)	0 (0)	1 (1.3)		6 (5.0)	0 (0)	6 (7.8)	
PR	29 (24.4)	5 (11.9)	24 (31.2)		41 (34.5)	10 (23.8)	31 (40.3)	
SD	85 (71.4)	34 (81.0)	51 (66.2)		69 (58.0)	29 (58.0)	40 (51.9)	
PD	4 (3.4)	3 (7.1)	1 (1.3)		3 (2.5)	3 (7.1)	0 (0)	
ORR	30 (25.2)	5 (11.9)	25 (32.5)	**0.014**	47 (39.5)	10 (23.8)	37 (48.1)	**0.010**
DCR	115 (96.6)	39 (92.9)	76 (98.7)	0.247	116 (97.5)	39 (92.9)	77 (100)	0.078

**Table 2** is a summary analysis of the best treatment effect according to RECIST1.1 and mRECIST criteria; complete response (CR), partial response (PR), stable disease (SD), disease progression (PD), ORR = CR + PR, DCR = CR + PR + SD; the value is expressed as the number of cases (percentage) or n (%); the P value is the calculation result of the bilateral chi-square test. Statistically significant results are highlighted in bold.

In the tumor response assessment across different subgroups, the ORR for the B7, B8-9, and treatment history subgroups were 26.4%, 23.4%, 25.7%, and 24.4%, respectively. In all subgroups, the ORR for patients receiving CT was higher than that of the IT group, although these differences were not statistically significant (all P > 0.05). Specifically, in the B7 group, the ORR for patients receiving CT (47.9% vs. 20.8%, P=0.026) was significantly higher than that of the IT group. Similarly, in patients with no prior treatment history, the ORR for the CT group (51.1% vs. 24.1%, P=0.01) was significantly higher than that of the IT group (both P < 0.05). However, in the B8-9 group and the subgroup with prior treatment history, the ORR was numerically higher for the CT group but did not reach statistical significance. Additionally, comparisons among subgroups indicated that patients in the B7 group had better tumor response outcomes than those in the B8-9 group, and patients with no prior treatment history had better outcomes than those with a history of antitumor treatment in the CP-B HCC cohort (in **Table 3**).

**Table 3:** Subgroup analysis of ORR.

Subgroup	RECIST 1.1				mRECIST			
	Total (n=119)	Intervention(n=42)	Combination(n=77)	P value	Total (n=119)	Intervention(n=42)	Combination(n=77)	P value
B7 vs B8-9				0.714				0.867
B7	19 (26.4)	3 (12.5)	16 (33.3)	0.059	28 (38.9)	5 (20.8)	23 (47.9)	0.026
B8-9	11 (23.4)	2 (11.1)	9 (31.1)	0.225	19 (40.4)	5 (27.8)	14 (48.3)	0.164
Treatment history				0.881				0.765
no	19 (25.7)	4 (13.8)	15 (33.3)	0.06	30 (40.5)	7 (24.1)	23 (51.1)	0.021
yes	10 (24.4)	1 (7.7)	10 (31.1)	0.199	17 (37.8)	3 (23.1)	14 (43.8)	0.195

**Table 3** shows the results of different subgroups according to RECIST 1.1 and mRECIST criteria; the values are expressed as the number of cases (percentage) or n (%); ORR (B7), ORR (B8-9), ORR (with treatment history), ORR (without treatment history) are the ORR of different subgroups and comparison; P value is the result of bilateral chi-square test calculation.

### 3.4 Cox regression results and survival prediction model

#### 3.4.1 OS

The univariate Cox regression model indicated that receiving more than three interventional treatments (HR=0.436, P=0.011) was associated with longer OS. Conversely, factors such as BCLC-C (HR=2.833, P=0.027), exceeding the up to 7 criteria (HR=2.304, P=0.017), tumor diameter over 5 cm (HR=2.194, P=0.017), PVTT (HR=1.889, P=0.047), and AFP over 400 ng/ml (HR=1.787, P=0.047) were linked to shorter survival. Furthermore, higher AFP levels correlated with increased survival risk. The multivariate Cox regression analysis revealed that combination therapy (P=0.003) and receiving more than three interventional treatments (HR=0.351, P=0.003) were independently associated with longer survival, while ascites (HR=2.776, P=0.004) was independently correlated with poorer survival prognosis (**Table 4**).

**Table 4:** OS of univariate and multivariate Cox regression.

Variables	Univariate		Multivariate	
	HR (95%CI)	P	HR (95%CI)	P
Sex (female VS male)	0.924 (0.413-2.064)	0.847		
Treatment (intervention VS combination)	1.783 (0.971-3.276)	0.062	2.911 (1.439-5.886)	**0.003**
Treatment history (yes VS no)	0.652 (0.344-1.235)	0.189		
Age (≥59 VS <59)	1.062 (0.599-1.883)	0.838		
CP score (B8-9 VS B7)	1.083 (0.606-1.935)	0.789		
BCLC (B VS A)	1.932 (0.782-4.773)	0.153	2.465 (0.944-6.438)	0.066
BCLC (C VS A)	2.833 (1.127-7.123)	**0.027**	1.809 (0.635-5.151)	0.267
Up To 7 (yes VS no)	2.304 (1.161-4.570)	**0.017**	1.372 (0.393-4.793)	0.620
Tumor diameter (>2 VS ≤2)	1.307 (0.725-2.355)	0.374		
Tumor diameter (>5cm VS ≤5cm)	2.194 (1.151-4.180)	**0.017**	2.787 (0.825-9.413)	0.099
Extrahepatic metastases (yes VS no)	1.460 (0.827-2.578)	0.192		
PVTT (yes VS no)	1.889 (1.007-3.544)	**0.047**	1.655 (0.653-4.194)	0.288
HBV/HCV (yes VS no)	0.634 (0.306-1.311)	0.219		
ALBI (2 VS 1)	0.934 (0.223-3.908)	0.925		
ALBI (3 VS 1)	3.510 (0.676-18.226)	0.135		
Ascites (yes VS no)	1.695 (0.944-3.044)	0.077	2.776 (1.380-5.582)	**0.004**
Times of intervention (>3 VS ≤3)	0.436 (0.231-0.825)	**0.011**	0.351 (0.174-0.706)	**0.003**
ECOG (1 VS 0)	1.195 (0.658-2.170)	0.559		
Cirrhosis (yes VS no)	0.844 (0.332-2.142)	0.721		
AFP (≥400ng/ml VS <400ng/ml)	1.787 (1.008-3.171)	**0.047**	1.921 (0.965-3.822)	0.063
NLR (≥3.0 VS <3.0)	1.163 (0.659-2.051)	0.603		
PLR (≥115.3 VS <115.3)	0.971 (0.550-1.712)	0.919		
Albumin (≥35g/L VS <35g/L)	1.133 (0.614-2.091)	0.689		

CP Score, Child-Pugh scoring system; BCLC, Barcelona Clinic Liver Cancer staging; up to 7, a criterion where the sum of the number of tumors (count) and the maximum tumor diameter (cm) exceeds 7; NLR, Neutrophil to Lymphocyte Ratio; PLR, Platelet to Lymphocyte Ratio; HR (Hazard Ratio), risk ratio; Multivariate COX, multivariate analysis conducted on factors with P < 0.1 from univariate Cox analysis. Statistically significant results are highlighted in bold.

#### 3.4.2 PFS

The univariate Cox regression model for progression-free survival (PFS) indicated that patients with PVTT (HR=2.237, P=0.008) had shorter PFS, while other factors showed no statistically significant differences (all P > 0.05). The multivariate Cox regression results indicated that patients receiving combination therapy had an independent association with prolonged PFS (P= 0.007). Additionally, treatment history (HR=1.939, P=0.048), presence of ascites (HR=3.696, P < 0.001), and AFP≥400 ng/ml (HR=2.002, P=0.036) were all independently associated with poor PFS outcomes (**Table 5**).

**Table 5:** PFS of univariate and multivariate Cox regression.

Variables	Univariate		Multivariate	
	HR (95%CI)	P	HR (95%CI)	P
Sex (female VS male)	0.987 (0.484-2.013)	0.971		
Treatment (intervention VS combination)	1.472 (0.834-2.598)	0.182	2.664 (1.306-5.430)	**0.007**
Treatment history (yes VS no)	1.467 (0.852-2.526)	0.167	1.939 (1.007-3.733)	**0.048**
Age (≥59 VS <59)	1.361 (0.796-2.325)	0.260		
CP score (B8-9 VS B7)	1.062 (0.624-1.806)	0.825		
BCLC (B VS A)	1.387 (0.649-2.965)	0.399		
BCLC (C VS A)	2.179 (0.984-4.824)	0.055		
Up To 7 (yes VS no)	1.452 (0.811-2.598)	0.210		
Tumor diameter (>2 VS ≤2)	1.305 (0.765-2.225)	0.328	1.711 (0.878-3.333)	0.115
Tumor diameter (>5cm VS ≤5cm)	1.591 (0.907-2.790)	0.105	1.832 (0.890-3.768)	0.100
Extrahepatic metastases (yes VS no)	1.036 (0.609-1.761)	0.897		
PVTT (yes VS no)	2.237 (1.238-4.042)	**0.008**	2.162 (1.074-4.349)	0.031
HBV/HCV (yes VS no)	0.704 (0.354-1.396)	0.315	0.451 (0.192-1.060)	0.068
ALBI (2VS 1)	0.873 (0.267-2.848)	0.821	0.814 (0.205-3.229)	0.770
ALBI (3VS 1)	2.609 (0.614-11.087)	0.194	3.289 (0.578-18.724)	0.180
Ascites (yes VS no)	1.584 (0.926-2.710)	0.093	3.696 (1.852-7.377)	**＜0.001**
Times of intervention (>3 VS ≤3)	1.046 (0.614-1.783)	0.868		
ECOG (1 VS 0)	1.361 (0.791-2.342)	0.266	1.689 (0.916-3.114)	0.093
Cirrhosis (yes VS no)	1.215 (0.483-3.061)	0.679	2.757 (0.845-8.999)	0.093
AFP (≥400ng/ml VS <400ng/ml)	1.540 (0.896-2.646)	0.118	2.002 (1.047-3.830)	**0.036**
NLR (≥3.0 VS <3.0)	1.115 (0.659-1.886)	0.686	1.810 (0.946-3.464)	0.073
PLR (≥115.3 VS <115.3)	1.190 (0.705-2.008)	0.515		
Albumin ( ≥35g/L VS <35g/L)	1.362 (0.772-2.405)	0.286	1.615 (0.821-3.176)	0.165

CP Score, Child-Pugh scoring system; BCLC, Barcelona Clinic Liver Cancer staging; up to 7, a criterion where the sum of the number of tumors (count) and the maximum tumor diameter (cm) exceeds 7; NLR, Neutrophil to Lymphocyte Ratio; PLR, Platelet to Lymphocyte Ratio; HR (Hazard Ratio), risk ratio; Multivariate COX, multivariate analysis conducted on factors with P < 0.1 from univariate Cox analysis. Statistically significant results are highlighted in bold.

#### 3.4.3 OS prediction model and nomogram

**Figure 4:**
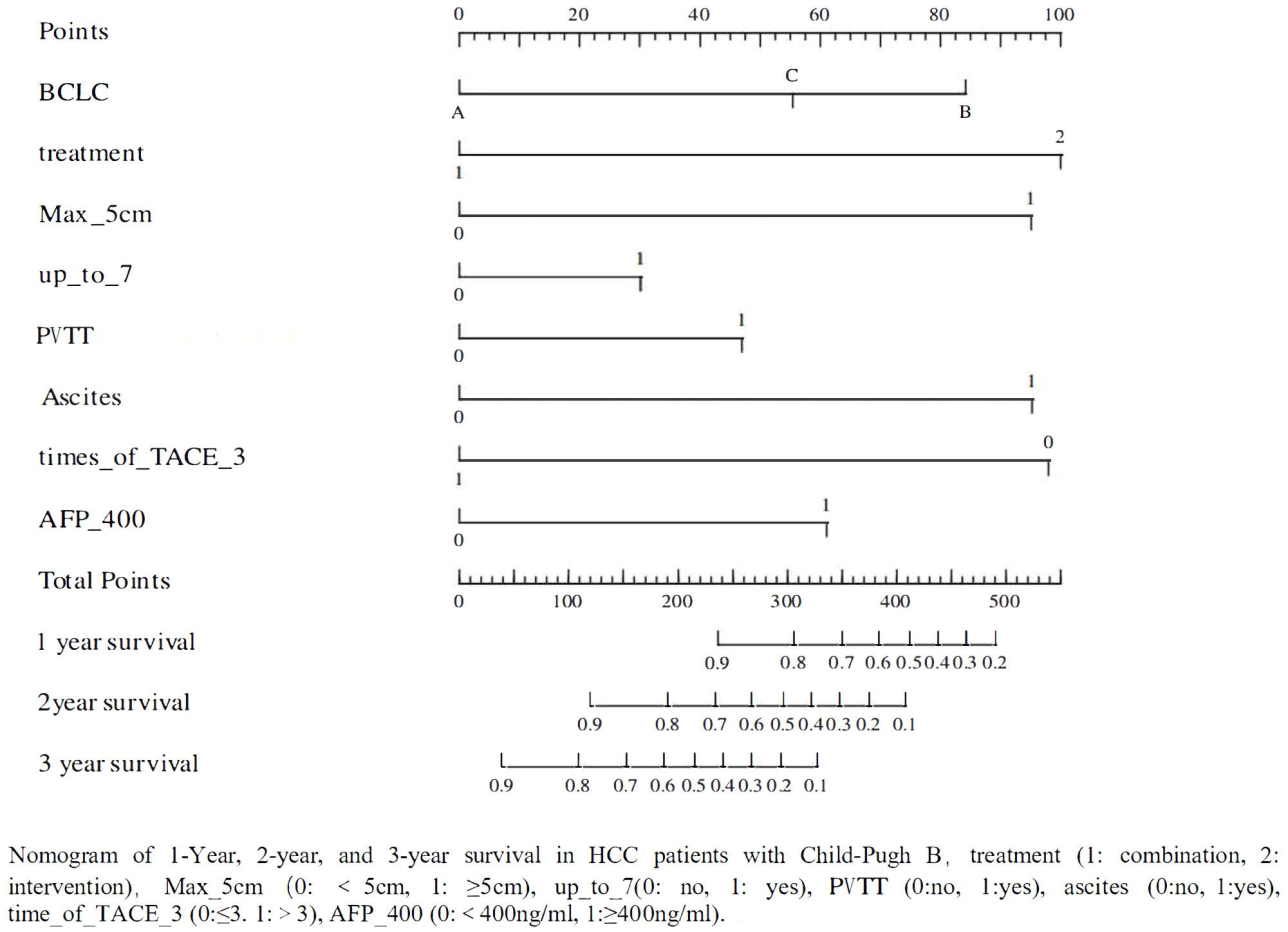
Nomogram of 1-Year, 2-year, and 3-year survival in HCC patients with Child-Pugh B.

A total of 119 patients with CP-B HCC were divided into training and validation sets in 7:3. Based on the results of univariate (P<0.1) and multivariate (P<0.05) Cox regression analyses for OS (**Table 4**), a nomogram was established to predict survival in CP-B HCC patients. The performance of the prediction model was evaluated using AUC curves, calibration curves, and decision curve analysis (17–19). The nomogram was developed based on the following eight factors: BCLC, treatment, tumor diameter, up to 7 criteria, presence of PVTT, ascites, times of interventional treatments, and AFP. The AUC for the 1-year survival prediction model was 0.82 (95% CI 0.72-0.92), and the AUC for the validation set was 0.79 (95% CI 0.63-0.96). The AUC for the 2-year survival prediction model was 0.89 (95% CI 0.80-0.98), while the AUC for the validation set was 0.77 (95% CI 0.56-0.98). The results from the nomogram indicated that both the 1-year and 2-year prediction models, as well as the validation sets, had AUC values greater than 0.7, demonstrating good predictive capability. The calibration curves showed high consistency between predicted and actual survival probabilities in the training cohort, although this consistency was less pronounced in the validation cohort, likely due to the smaller sample size (**Figure 4**). However, the AUC for the 3-year prediction model was 0.88 (95% CI 0.77-0.99), with an AUC of 0.69 (95% CI 0.32-1.07) for the validation set, indicating that the nomogram had limited predictive ability for 3-year survival rates. The calibration curves also showed poor consistency between the training and validation cohorts for this time frame (**Supplementary Figures 1**, **2**).

### 3.5 Subgroup analysis

**Figure 5:**
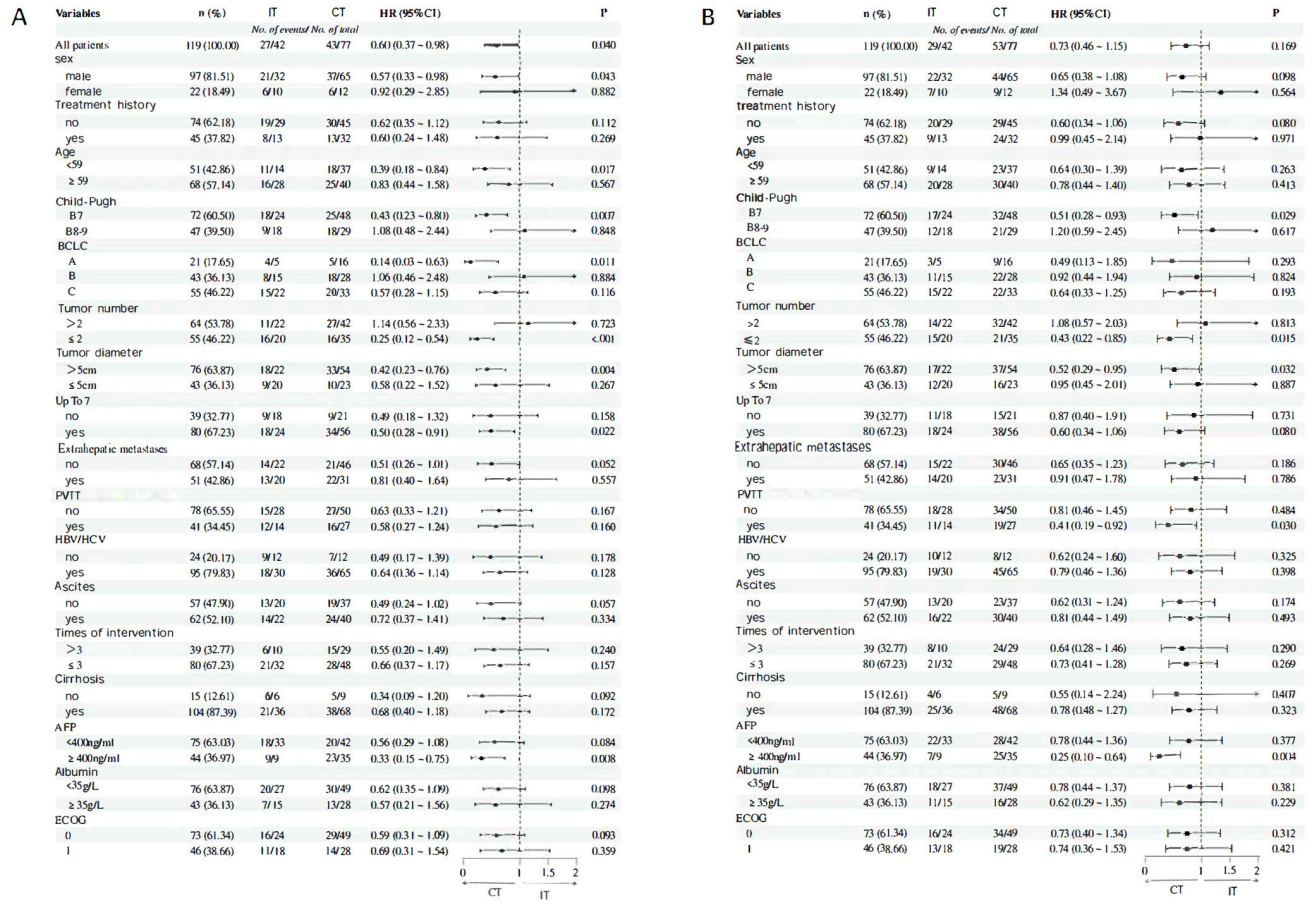
**(A)** OS subgroup analysis and forest flot **(B)** Results of PFS subgroup analysis and forest flot.

In univariate Cox regression subgroup analysis comparing different treatment regimens (IT vs. CT), the results indicated that combination therapy was associated with prolonged OS in the following subgroups: male patients (HR=0.57, P=0.043), those aged <59 years (HR=0.39, P=0.017), B7 stage (HR=0.43, P=0.007), BCLC A (HR=0.14, P=0.011), patients with ≤2 tumors (HR=0.25, P < 0.001), tumors with a diameter >5 cm (HR=0.42, P=0.004), those exceeding the up to 7 criteria (HR=0.50, P=0.022), and AFP ≥400 ng/ml (HR=0.33, P=0.008) (**Figure 5A**). In other subgroups, the P-values were all greater than 0.05, indicating that combination therapy did not provide a significant survival benefit compared to interventional treatment. Subgroups associated with improved PFS due to combination therapy included B7 stage (HR=0.51, P=0.029), patients with ≤2 tumors (HR = 0.43, P = 0.015), tumors with a diameter >5 cm (HR=0.52, P=0.032), those with portal vein tumor thrombus or vascular invasion (HR=0.41, P=0.030), and AFP ≥400 ng/ml (HR=0.25, P=0.004) (**Figure 4**). In other subgroups, P-values were >0.05, suggesting that combination therapy did not result in longer PFS (**Figure 5B**).

### 3.6 Adverse event analysis

Among the 119 liver cancer patients, related adverse events (rAEs) occurred during treatment. A total of 52 patients (43.7%) experienced leukopenia, with 15 patients (12.6%) classified as grade 3 or higher. Additionally, 90 patients (75.6%) experienced thrombocytopenia, with 31 patients at grade 3 or higher. Prolonged PT was observed in 29 patients (24.4%); 22 patients (18.5%) had elevated ALT, with 5 at grade 3 or higher; 45 patients (37.8%) experienced elevated AST, with 10 patients at grade 3 or higher; 59 patients (49.6%) had elevated TBIL, with 8 patients at grade 3 or higher; 26 patients (21.8%) developed hypothyroidism; 21 patients (17.6%) showed further progression in their Child-Pugh scores, and 5 patients (4.2%) experienced progression in their ALBI scores.

The incidence of adverse events, ranked from highest to lowest, was as follows: thrombocytopenia, elevated total bilirubin, leukopenia, elevated AST, prolonged PT, hypothyroidism, elevated ALT, progression of Child-Pugh scores, and progression of ALBI scores. With the exception of elevated AST, the incidence of other adverse events was higher in the CT group compared to the IT group, with particularly notable differences observed in hypothyroidism and progression of Child-Pugh scores. However, for all other adverse events, no significant statistical differences were found between the two groups, with P-values all exceeding 0.05 (**Table 6**).

**Table 6:** Full grade and ≥grade 3 adverse events.

Adverse event	Total (n=119)	Full grade			Total (n=119)	≥grade 3		
		IT (n=42)	CT (n=77)	P		IT (n=42)	CT (n=77)	P
Decreased platelet count	90 (75.6)	30 (71.4)	60 (77.9)	0.430	31 (26.1)	10 (23.8)	21 (27.3)	0.681
Elevated TBIL	59 (49.6)	17 (40.5)	42 (54.5)	0.142	8 (6.7)	3 (7.1)	5 (6.5)	0.892
Decreased white blood cell count	52 (43.7)	14 (33.3)	38 (49.3)	0.092	15 (12.6)	6 (14.3)	9 (11.7)	0.683
Elevated AST	45 (37.8)	19 (45.2)	26 (33.8)	0.219	10 (8.4)	6 (14.3)	4 (5.2)	0.088
PT extension	29 (24.4)	7 (16.7)	22 (28.6)	0.148				
hypothyroidism	26 (21.8)	5 (11.9)	21 (27.3)	0.053				
Elevated ALT	22 (18.5)	6 (14.3)	16 (20.8)	0.383	5 (4.2)	3 (7.1)	2 (2.6)	0.482
Progression of the Child-Pugh Score	21 (17.6)	1 (2.4)	20 (26.0)	**0.003**				
Progress in ALBI grading	5 (4.2)	1 (2.4)	5(5.2)	0.800				

Expressed as frequency and percentage i.e. n (%); PT, prothrombin time; ALT alanine aminotransferase; AST, azelaic aminotransferase; ALBI classification: albumin-bilirubin score. Statistically significant results are highlighted in bold.

## 4 Discussion

This retrospective study aimed to investigate the efficacy and safety of transarterial interventional treatments (including TACE and HAIC) with or without targeted therapy in patients with CP-B HCC. The results confirmed that patients with CP-B HCC who received combination therapy had a longer OS compared to those who underwent interventional treatment (21.4 months vs 13.2 months), with statistical significance. However, there was no significant improvement in PFS, although numerically, combination therapy still outperformed interventional treatment (12.7 months vs 10.9 months). Similarly, combination therapy demonstrated a clear advantage in ORR (RECIST 1.1: 32.5% vs 11.9%; mRECIST: 40.3% vs. 23.8%). Therefore, even for CP-B HCC patients, combination therapy remains the preferred option when it can be tolerated. The findings were particularly consistent for the B7 subgroup, where the results showed a significant advantage for combination therapy in OS (24.6 months vs 11.9 months), PFS (14.8 months vs 8.6 months), and ORR. In contrast, the B8-9 subgroup did not show significant differences in these outcomes. Notably, while combination therapy did not demonstrate a clear advantage in OS for the B8-9 group, it still numerically outperformed interventional treatment (20.9 months vs 13.2 months). On the other hand, there was no apparent difference in survival benefits between the B7 and B8-9 groups, suggesting that the limited sample size of this study may have contributed to the lack of observed differences.

The Guidelines for the Diagnosis and Treatment of Primary Liver Cancer (2022 version) (20) indicate that TACE is an important treatment option for HCC patients with CP-B liver function. After careful assessment of liver function reserve and tumor burden, TACE can effectively control tumor progression by embolizing the tumor’s blood supply and locally administering chemotherapy, leading to ischemic necrosis of the tumor. A multicenter retrospective study on TACE treatment reported (21) the mOS of 11.5 months and the mPFS of 6.2 months, which, although lower than that of CP-A stage patients, still reflects a notable survival benefit. Despite the efficacy of TACE, limitations remain for patients with CP-B HCC. Systemic therapies have shown promising data in HCC patients (22–24), but limitations persist for those with CP-B liver function. ICIs have been widely applied in the treatment of hepatocellular carcinoma by blocking the PD-1/PD-L1 pathway, thereby reactivating T-cell immune responses against tumors. The CheckMate 040 study (25) provided the first prospective clinical report on immunotherapy for CP-B stage HCC patients, revealing the ORR of 12% and the DCR of 55%, with acceptable safety profiles comparable to those of CP-A patients. Professor Fan-pu Ji’s team (26) conducted a meta-analysis of 22 published studies, finding that the ORR for advanced HCC patients with CP-B stage receiving ICIs was 14%, with a DCR of 46%, mOS of 5.5 months, and mPFS of 2.7 months. Targeted therapies, including vascular endothelial growth factor (VEGF) and its receptor (VEGFR) inhibitors, as well as multi-kinase inhibitors (MKIs), interfere with tumor cell growth, proliferation, metastasis, and survival, providing some survival benefits for HCC patients. The GIDEON study (27) reported an mOS of 5.2 months for sorafenib in CP-B HCC patients, which is lower than the 13.6 months observed in CP-A patients (28). However, subgroup analysis in the Chinese population indicated good efficacy and safety. Lenvatinib has also demonstrated certain efficacy and safety in similar populations (29, 30). Thus, while single-agent interventional, targeted, or immunotherapy may be superior to best supportive care, the survival and tumor response data do not show a significant enhancement (31).

In the exploration of combination therapy, the EMERALD-1 study (32) demonstrated that as the first global phase III trial, the immunotherapy-targeted therapy combined with TACE (including durvalumab and bevacizumab) significantly improved PFS in patients with unresectable HCC suitable for embolization. The PFS for patients receiving this combination therapy was 15.0 months, with an ORR of 43.6%; notably, 98% of these patients were classified as CP-A, while only 2% were CP-B. In the CHANCE series of studies, the CHANCE-001 trial (33) showed that patients undergoing TACE combined with PD-(L)1 inhibitors and targeted therapies had PFS (mRECIST) of 9.5 months compared to 8.0 months in the TACE monotherapy group (P=0.002), demonstrating a significant improvement. The ORR (mRECIST) was 60.1%, and the overall survival (OS) was 19.2 months, with a majority of the enrolled patients (83%) classified as CP-A. The CHANCE-2211 study (14) analyzed patients with advanced liver cancer who had previously received TACE combined with carelizumab and apatinib, reporting significant improvements in OS (24.1 months vs 15.7 months), PFS (13.5 months vs 7.7 months), and ORR (59.5% vs 37.4%), with 85% of participants classified as CP-A. In the high-evidence CHANCE-2201 study (12), data were processed using a treatment-weighted inverse probability method (sIPTW) to minimize the influence of confounding factors, resulting in findings that closely resemble those of randomized controlled trials (RCTs). This study supports the use of TACE combined with ICIs and anti-VEGF antibodies/TKIs as first-line treatment for advanced HCC, showing significant improvements in OS (22.6 months vs 15.9 months), PFS (9.9 months vs 7.4 months), and ORR (RECIST 1.1: 41.2% vs. 22.9%; mRECIST: 47.3% vs. 29.7%), with 82.2% of patients classified as Child-Pugh A. These studies collectively indicate that combination therapies involving interventional treatment with targeted and immune therapies yield longer OS and PFS compared to single-agent interventional or targeted-immune combination therapies, with safety profiles remaining within acceptable limits. However, the majority of the enrolled patients were CP-A, with a limited representation of CP-B patients, particularly those with Child-Pugh scores of B8-9. This study focuses on the CP-B HCC population with poorer liver function, addressing a significant gap in treatment data for this group. Currently, there are no clear definitions for the selection criteria for arterial interventional therapy and systemic treatment in CP-B HCC patients, nor is there a definitive assessment of their associated risks (34). Poorer liver function may be a crucial factor limiting treatment options, as the liver damage caused by interventional therapies and systemic treatments combining targeted and immune therapies could exacerbate liver function deterioration in B8-9 HCC patients, potentially diminishing treatment efficacy.

In the multivariate COX regression analysis, it was found that the choice of combination therapy is a favorable factor for improving OS and PFS. Additionally, an increased number of interventional treatments was more likely associated with longer OS, which aligns with previous clinical research findings (13). Notably, the presence of ascites was identified as an independent risk factor affecting both OS and PFS. Ascites can reduce puncture accuracy, lead to abnormal distribution of embolic agents and chemotherapy drugs at the tumor site, and increase the risk of puncture-related complications, thereby impacting the effectiveness of interventional therapy (35). Therefore, the volume of ascites is an important assessment factor when considering whether CP-B HCC patients can undergo interventional treatment (36, 37). Furthermore, the nomograms created based on univariate and multivariate COX regression results indicate that, aside from the choice of treatment regimen, other predictive factors are closely related to liver function (particularly ascites) and tumor burden (such as tumor diameter, up to 7 criteria, PVTT, and AFP).

In conducting subgroup analyses for precise treatment selection for specific populations, the characteristics of patients who benefited from longer OS with combination therapy included: male gender, age<59, CP score of B7, tumors exceeding the up-to-7 criteria, tumor count of ≤2, tumor diameter >5 cm, and AFP ≥400 ng/ml. The population likely to improve PFS with combination therapy included patients with a CP score of B7, tumor number ≤ 2, tumor diameter >5 cm, and the presence of PVTT. Thus, it can be hypothesized that among CP-B HCC patients, those with relatively better liver function but greater tumor burden is more likely to benefit from combination therapy, whereas other subgroups may not experience improvements in OS or PFS from combination treatment.

The safety of anti-tumor drugs is also a critical consideration; if a patient cannot tolerate the adverse effects of these medications, further exploration of treatment options would be meaningless. We observed that common adverse events included thrombocytopenia, increase TBIL, leukopenia, and increased AST levels, with no significant increase in these events within the CT group. This is consistent with the findings from most retrospective studies on interventional combined targeted therapies (12–15). Notably, the occurrence of worsening CP scores during combined treatment was significantly higher than that associated with interventional treatment alone, suggesting a greater risk of further liver function deterioration among CP-B HCC patients. Previous studies have indicated that initial TACE treatment can lead to an immediate deterioration of liver function from CP-A to CP-B in newly diagnosed HCC patients (38).

The risk of liver function deterioration becomes a major factor influencing survival benefits and treatment options for CP-B HCC patients, as well as a commonly occurring event. Both local anti-tumor therapies and systemic treatments can exacerbate liver failure or even increase mortality risk. The liver, being a crucial organ for the metabolism and clearance of most anti-cancer drugs, sees reduced activity of cytochrome P450 enzymes and other drug-metabolizing enzymes when liver function is compromised, leading to decreased drug clearance rates and potentially diminished treatment efficacy. Additionally, diminished detoxification functions and reduced protein synthesis can hinder the transport and distribution of drugs in the body, increasing the toxicity of anti-tumor medications (39, 40). Consequently, this may not only fail to achieve tumor treatment objectives but could also further impair liver function. In the process of tumor treatment, it is essential to balance managing liver diseases, such as cirrhosis, and reducing tumor burden to delay the progression of liver failure and improve long-term survival rates (7). Accurate assessment of liver function is crucial. A retrospective study (41) has also suggested that both ALBI grading and Child-Pugh scores are independent factors influencing patient prognosis, with ALBI scoring being more objective in nature. Thus, precise evaluation of liver function is key to selecting appropriate treatment regimens for these patients, particularly for those with a Child-Pugh score of B8-9, who should carefully consider risks associated with combined treatment to avoid exacerbating liver insufficiency.

Most studies center on CP-A HCC, leaving scant data for CP-B patients’ treatment. Their disease complexity and lack of clinical data hamper doctors’ treatment choices, and empirical treatments often fall short. This study evaluates interventional, targeted, and immunotherapies for HCC with liver dysfunction, aiming to offer data for precise CP-B treatment. It explores treatment impacts on CP-B HCC, aids clinicians in choosing suitable plans per patients’ conditions, and promotes precision in liver cancer treatment. Rizzo (42, 43) noted HCC immunotherapy has challenges as the tumor microenvironment complexity may hinder anti-tumor effects. Biomarkers can predict drug efficacy, and studying resistance mechanisms helps. Interventional - ICIs combination is emerging in advanced HCC treatment, to be more widely used in 5 years. Precise patient selection via better understanding of cancer mechanisms and genetic testing by artificial intelligence (AI) aided analysis, will lead to advanced diagnostic models for personalized HCC treatment.

However, this study has limitations: 1) Data bias from loss to follow - up or treatment inaccessibility; 2) Coarse grouping due to variable treatment intervals and imaging methods; 3) Single - center, small - sample retrospective design lacking strong evidence. Larger multi - center prospective studies are needed for better guidance in treating moderate liver-dysfunction patients.

## 5 Conclusion

Under controllable liver function impairment, the combination of interventional therapy with targeted and immunotherapy is a safe and effective option for CP-B HCC patients. Ascites impacts survival and treatment. More interventional treatments may improve survival.

## Data Availability

The raw data supporting the conclusions of this article will be made available by the authors, without undue reservation.
