# Supplementary material for: Comparative efficacy of interventional therapy with or without targeted immunotherapy in Child-Pugh B hepatocellular carcinoma patients: a single-center, retrospective study
Source: Front Oncol. 2025 May 1;15:1541805. doi: 10.3389/fonc.2025.1541805 (PMC12078163; doi:10.3389/fonc.2025.1541805)
Supplement: Supplementary file 1 [file DataSheet1.docx]

**Supplementary Material**


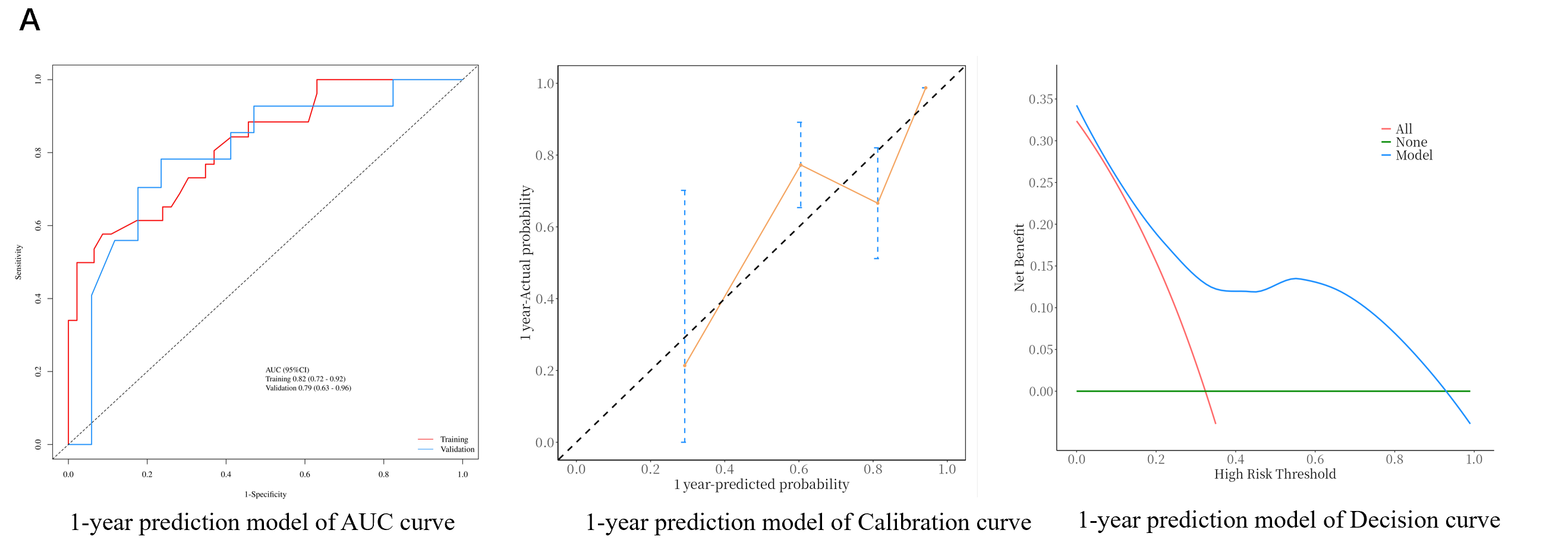


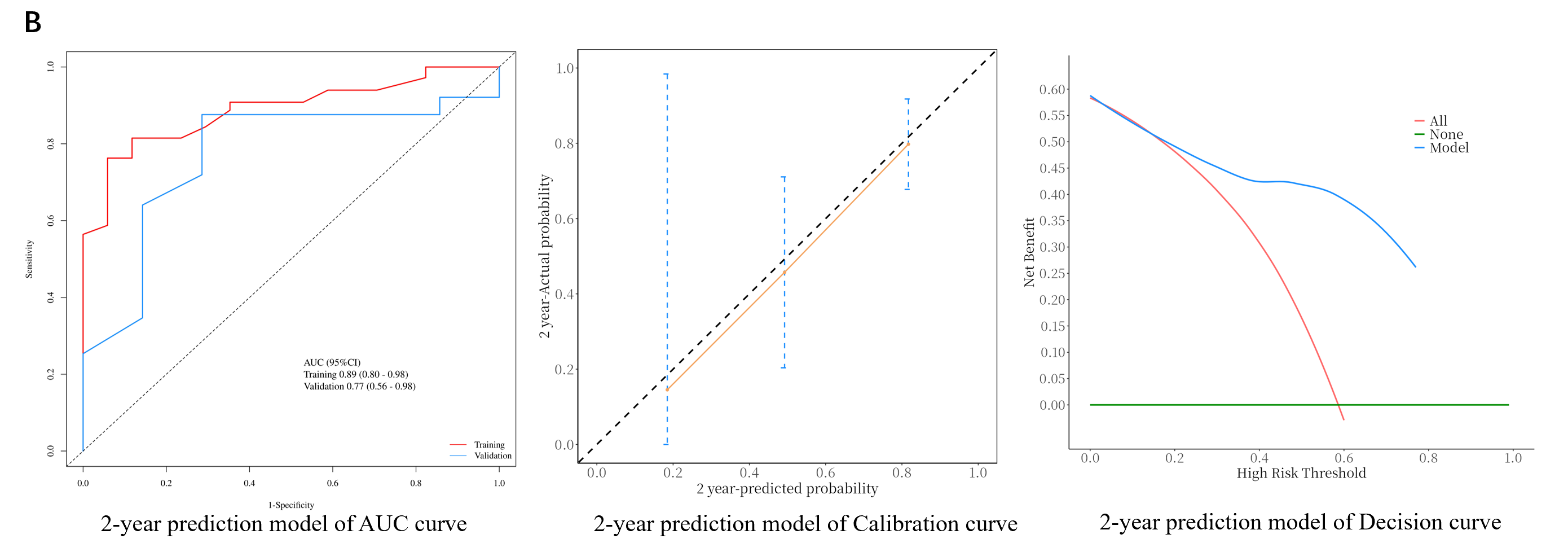

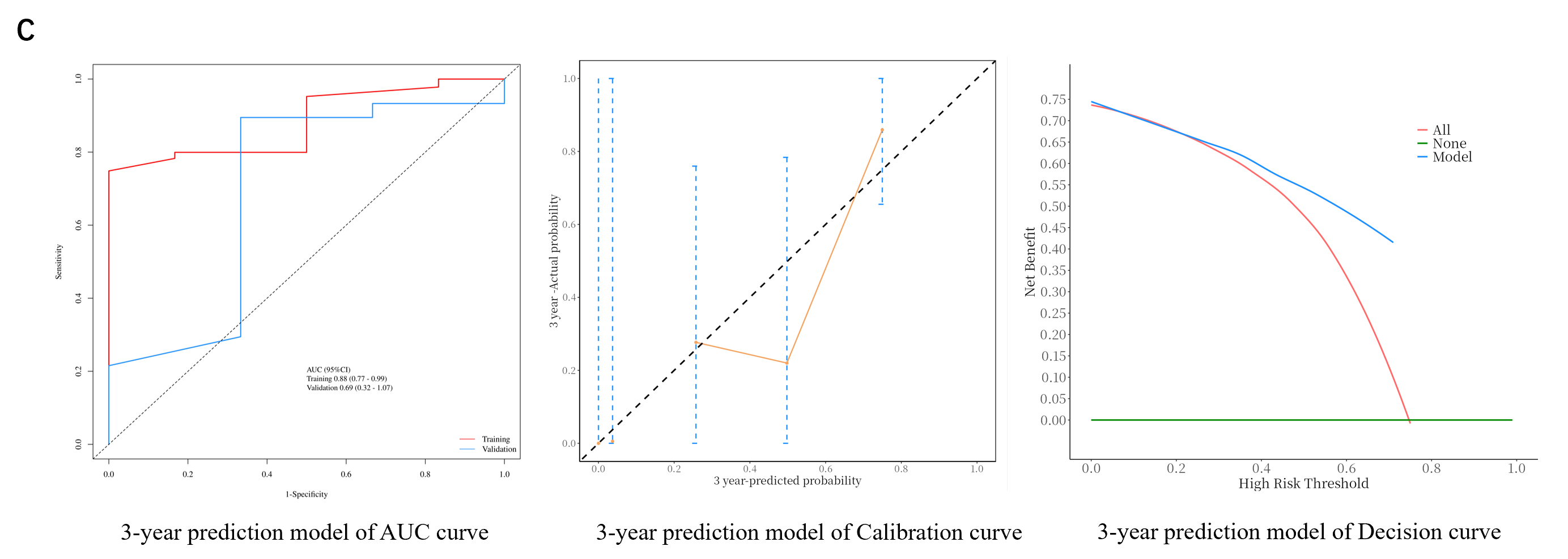


**Supplementary Figure 1**. A. 1-yearprediction of AUC curve-Training: 0.82 (95%CI 0.72-0.92）; Validation: 0.79 (95%CI 0.63-0.96); 1-yearprediction of Calibration curve-Training; 1-yearprediction of Decision curve-Training; B. 2-yearprediction of AUC curve-Training: 0.89 (95%CI 0.80-0.98); validation: 0.77 (95%CI 0.56-0.98); 2-yearprediction of Calibration curve-Training; 2-yearprediction of Decision curve-Training; C. 3-yearprediction of AUC curve-Training: 0.88 (95%CI 0.77-0.99); Validation: 0.69 (95%CI 0.32-1.07); 3-yearprediction of Calibration curve-Training; 3-yearprediction of Decision curve-Training.


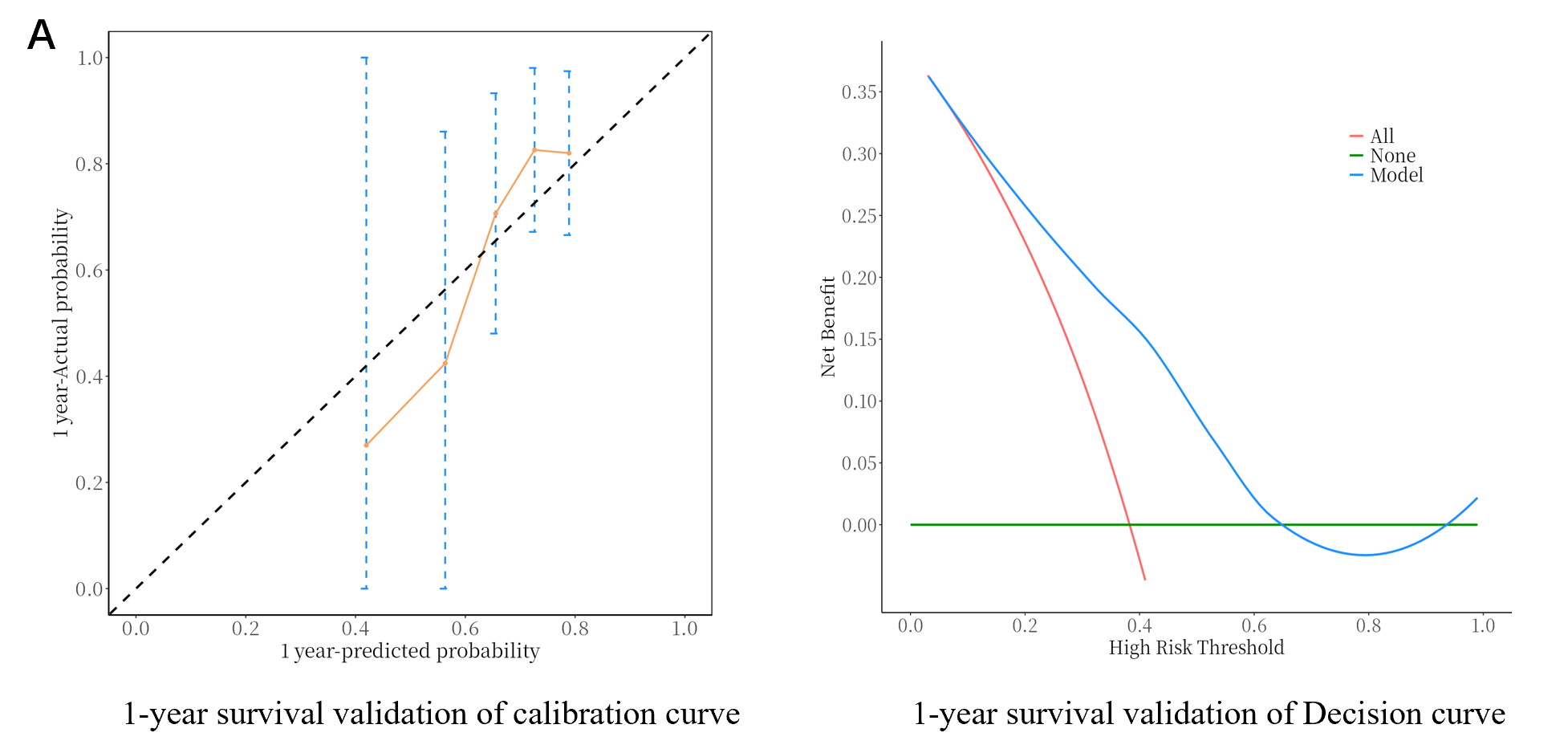

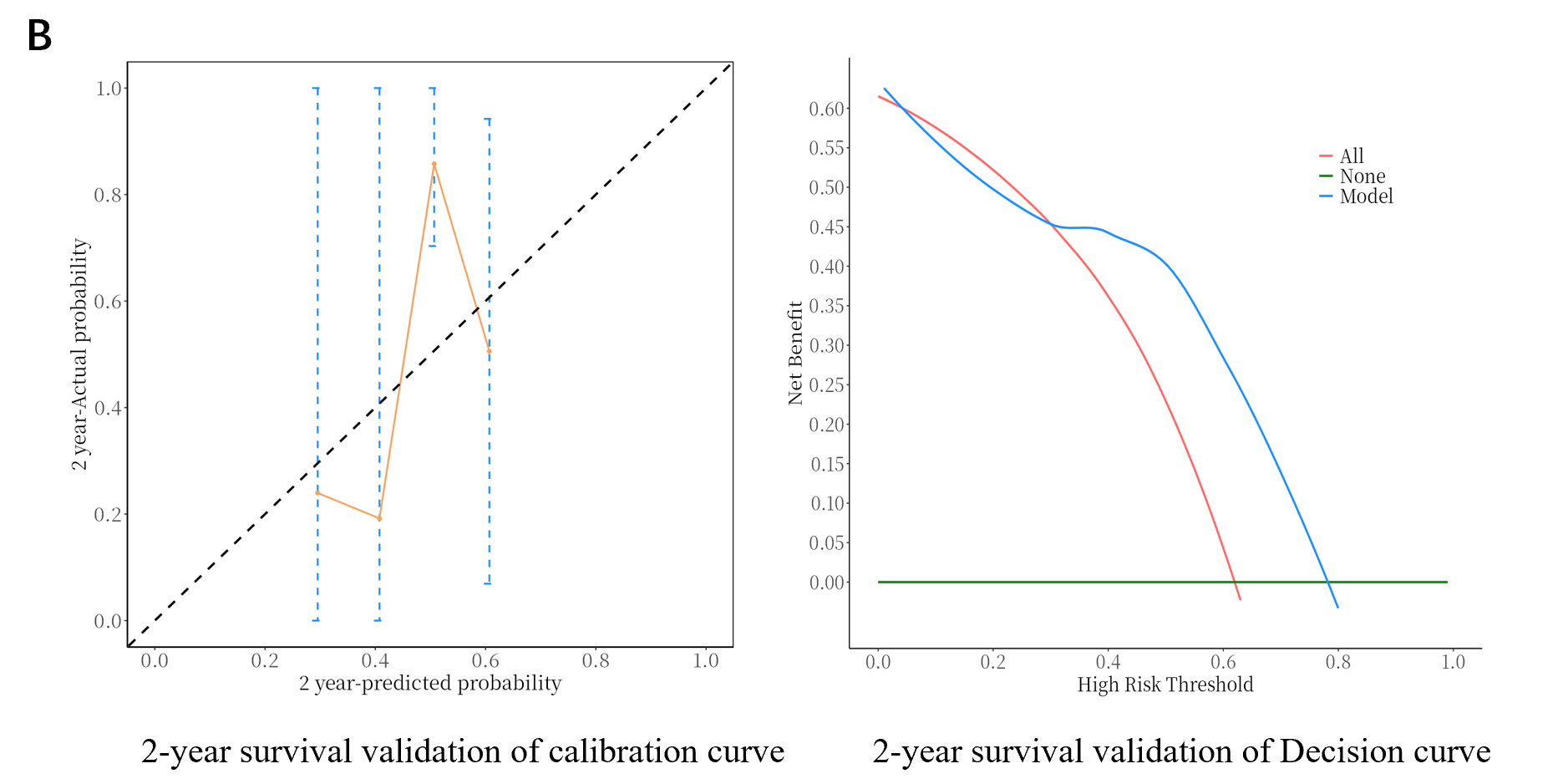

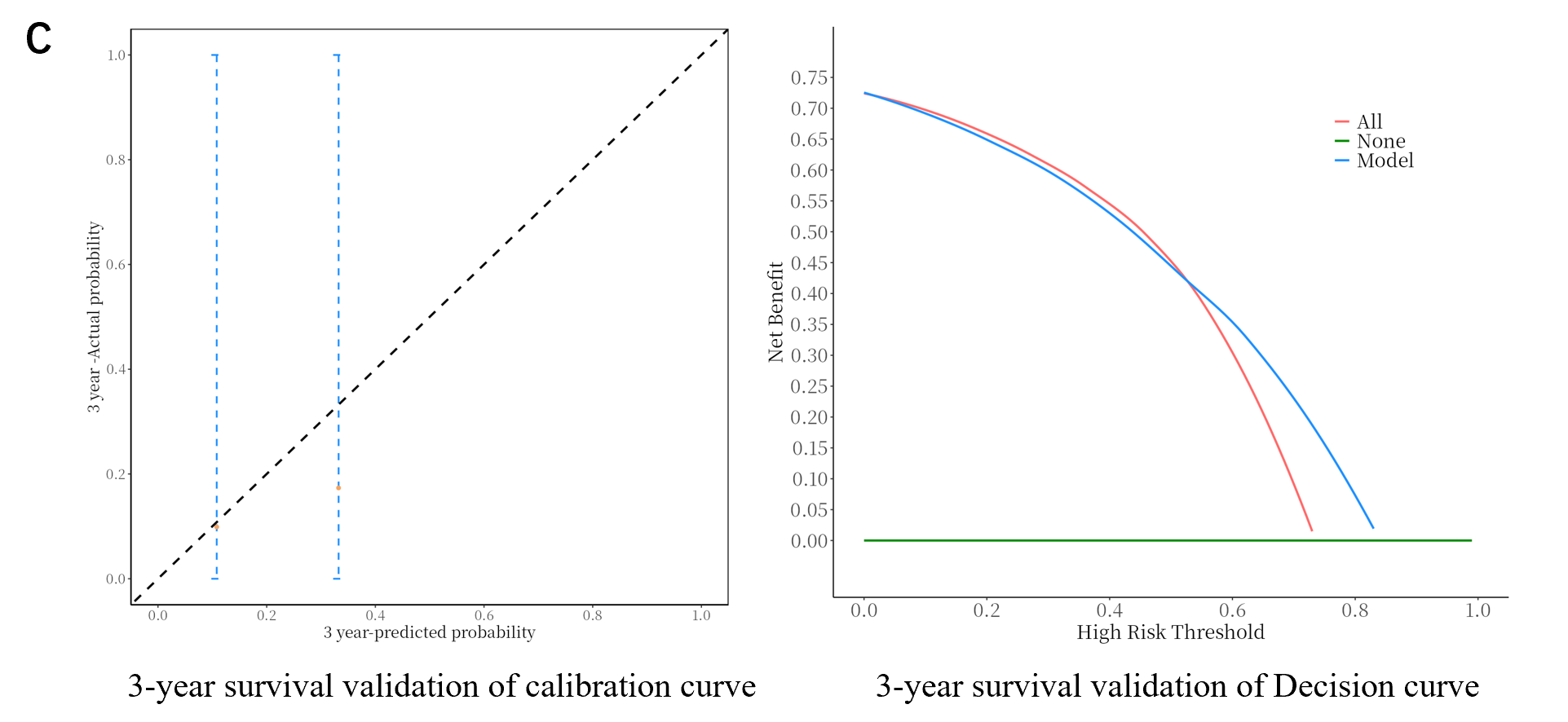


**Supplementary Figure 2**. A. 1-year prediction of Calibration curve-validation; 1-yearprediction of Decision curve-validation; B. 2-yearprediction of Calibration curve-validation; 2-yearprediction of Decision curve-validation; C. 3-yearprediction of Calibration curve-validation; 3-yearprediction of Decision curve-validation.
